# Protective role of serum albumin in dementia: a prospective study from United Kingdom biobank

**DOI:** 10.3389/fneur.2024.1458184

**Published:** 2024-08-14

**Authors:** Yiyuan Cui, Chunyu Li, Bin Ke, Yi Xiao, Shichan Wang, Qirui Jiang, Xiaoting Zheng, Junyu Lin, Jingxuan Huang, Huifang Shang

**Affiliations:** Department of Neurology, Laboratory of Neurodegenerative Disorders, National Clinical Research Center for Geriatrics, West China Hospital, Sichuan University, Chengdu, China

**Keywords:** albumin, microalbumin, neurodegenerative disorders, dementia, United Kingdom biobank

## Abstract

**Background:**

A number of studies have explored the link between neurodegenerative disorders (NDDs) and albumin, the main protein in human plasma. However, the results have been inconsistent, highlighting the necessity for a detailed systemic analysis.

**Methods:**

Utilizing data from the United Kingdom Biobank, we investigated the relationship between baseline levels of serum and urine albumin and the occurrence of common NDDs, including Parkinson’s disease (PD), amyotrophic lateral sclerosis (ALS) and dementia, employing Cox proportional hazards regression analysis.

**Results:**

Our results reveal that elevated baseline serum albumin levels are linked to a decreased risk of developing dementia (beta = −0.024, SE = 0.004, *p* < 0.001). Subgroup and interaction analyses highlighted the impact of factors like body mass index (BMI), age, and alcohol consumption on this relationship. Specifically, participants with higher BMI, younger age, or lower alcohol intake exhibited a stronger protective effect. On the other hand, a higher baseline level of urine microalbumin was connected to a slight increase in dementia risk (beta = 0.003, SE = 3.30E-04, *p* < 0.001). No significant associations were found between albumin levels and the risk of PD or ALS.

**Conclusion:**

Our study underscores the potential role of serum albumin as a biomarker associated with reduced dementia risk. These findings contribute valuable insights into the understanding of albumin’s impact on NDDs, suggesting its utility as a biomarker for dementia in clinical settings and informing future therapeutic strategies in clinical trials.

## Introduction

Neurodegenerative disorders (NDDs) comprise a diverse range of conditions marked by the progressive decline of neuronal structure and function (1). While current treatments primarily address symptom management, there is a pressing need for timely and precise diagnostic methods to inform the development of therapies aimed at decelerating disease progression and improving patient outcomes (2). Notably, the pathogenesis of NDDs often begins years before clinical symptoms become evident. By the time these symptoms appear, significant neuronal and synaptic damage has already taken place. Early detection of individuals in the pre-symptomatic phase through biomarkers is crucial for both primary and secondary prevention trials. This will also enable the efficient development and evaluation of disease-modifying treatments, particularly during the early stages of the disease (3, 4).

Blood-based biomarkers represent a groundbreaking advancement in the clinical evaluation of NDDs, offering the potential for early disease detection before symptoms emerge (5, 6). Among these biomarkers, albumin, the primary plasma protein synthesized in the liver, plays a crucial role in the pathogenesis of NDDs. As a carrier protein, albumin facilitates the stable transport of hydrophobic and hydrophilic molecules, including free fatty acids, steroid hormones, medications, and metal ions. Additionally, it functions as a potent antioxidant, significantly contributing to the overall antioxidant capacity of plasma, thus positioning serum albumin as a critical factor in neurodegeneration. Epidemiological studies have highlighted correlation between albumin level and NDD risk. For instance, a comprehensive seven-year retrospective cohort study involving 1,800 subjects demonstrated that reduced serum albumin concentrations independently raise the risk of mild cognitive impairment (MCI) in older adults (7), though another study found that serum albumin concentrations did not differ according to cognitive function in 540 older cognitively normal adults from the single-center Conselice Study of Brain Aging (8). Meanwhile, previous research indicates that low serum albumin levels may exacerbate amyloid accumulation, thereby increasing the risk of Alzheimer’s disease (AD) dementia (9), consistent with another cohort study which found that high level of serum albumin was associated with low risk of cognitive impairment among individuals aged ≥65 years (10). Additionally, lower serum albumin levels at the time of amyotrophic lateral sclerosis (ALS) diagnosis were linked to a higher mortality risk (11), and another study reported increased survival in ALS patients with higher serum albumin levels (12). A cross-sectional study involving 96 Parkinson’s disease (PD) patients and 108 healthy controls found that serum albumin levels were significantly decreased in PD patients and were identified as independent risk factors for PD (13). Meanwhile, higher serum albumin levels have also been associated with better cognitive function and proposed to offer protection against severe motor impairment and PD-related mortality (14). Collectively, these findings emphasize albumin’s crucial role in the pathogenesis of NDDs. However, it is essential to acknowledge potential limitations of observational studies, such as small sample sizes and confounding variables. Additionally, since these studies predominantly involved prevalent cases, further research is needed to determine whether prediagnostic albumin levels reliably predict future NDD risks.

In this study, we examined the relationship between baseline levels of serum and urine albumin and the risk of common NDDs using longitudinal data from the United Kingdom (Uniterd Kingdom) Biobank. Our findings indicate that individuals with higher baseline serum albumin levels exhibited a significantly lower risk of developing incident dementia.

## Methods

### Participants

The study utilized data from the United Kingdom Biobank, a large-scale prospective cohort study that enrolled approximately 500,000 United Kingdom residents aged 39 to 72 years between 2006 and 2010 (15). All participants provided informed consent for data collection and linkage. The current research utilized the United Kingdom Biobank resource under application number 98992.

### Exposures

Participants’ baseline serum albumin levels were the primary exposure of interest in this study. Blood samples were collected from participants during the final stage of their baseline visit and subsequently stored at −80°C until analysis. Serum albumin levels were measured using enzymatic analysis on a Beckman Coulter AU5800 machine, utilizing a kinetic modification of the Jaffe procedure. Detailed information on the assay methodology and quality control protocols can be found on the United Kingdom Biobank website.^1^ Additionally, baseline urine microalbumin levels were analyzed as a secondary exposure. Urine samples were collected at baseline, and urine albumin measurements were performed using the Beckman Coulter AU5400 clinical chemistry analyzer with the manufacturer’s designated reagents and calibrators.

### Outcomes

Our primary outcome of interest was the incidence of newly diagnosed common NDDs, including ALS, PD, and all-cause dementia. Diagnostic codes for these conditions were based on the International Classification of Diseases, Tenth Revision (ICD-10) and the Read coding system (Supplementary Table S1). Follow-up for NDD incidence continued until November 9, 2022. The baseline period was defined as the date of participant recruitment, and the follow-up period ended with the date of NDD diagnosis, date of death, or the conclusion of the follow-up period, whichever occurred first. To address concerns about reverse causality, participants with prevalent NDDs at the time of enrollment were excluded. Additionally, individuals with a latency period of 2 years or less between initial sampling and NDD diagnosis were also excluded to reduce the potential influence of reverse causation.

### Statistical analysis

We employed Cox proportional hazards models to evaluate the associations between baseline albumin levels and the risk of each NDD, calculating hazard ratios (HRs) and 95% confidence intervals (CIs). Initial analyses were performed on the entire study population, followed by stratification based on demographic factors such as gender (males and females), age (<65 years and ≥ 65 years), and BMI (<25 kg/ m^2^ and ≥ 25 kg/ m^2^). Interaction analyses were conducted by including interaction terms in the Cox models. Two models were used for the analyses. Model 1 included adjustments for basic demographic variables, such as sex and age, which can influence NDD incidence. Model 2, the fully adjusted model, incorporated additional covariates to account for socioeconomic status (e.g., Townsend deprivation index, education) and lifestyle factors (e.g., BMI, smoking status, alcohol consumption; Supplementary materials). Participants with missing values for any variables included in the models were excluded to maintain the robustness and accuracy of the results. All statistical analyses were performed using R v3.5.3.

## Results

### Population characteristics

**Figure 1:**
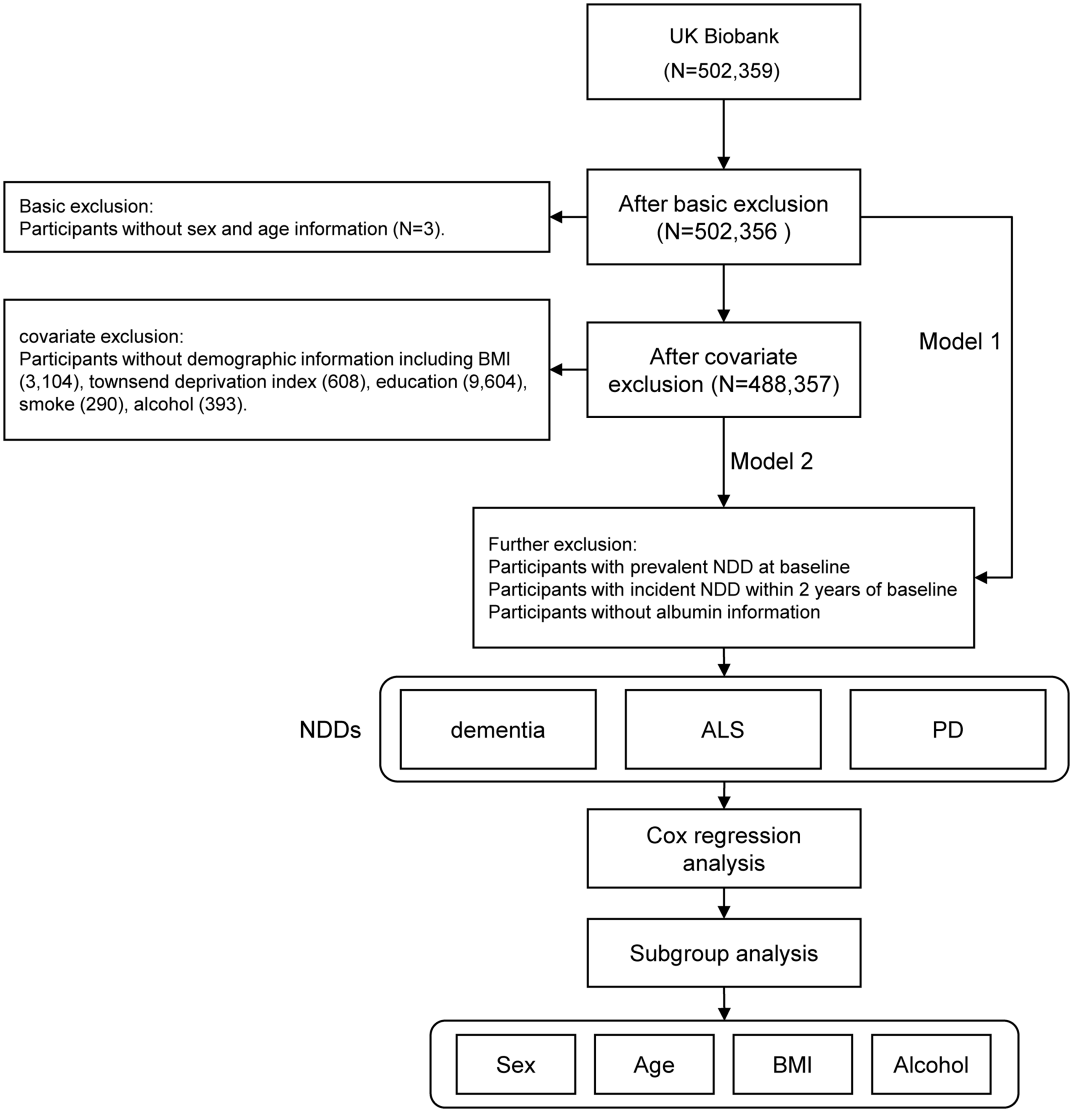
Schematic overview of the study design.

The United Kingdom Biobank initially recruited 502,359 participants. After excluding individuals with missing age information (*N* = 3) and those without sex information (*N* = 0), a total of 502,356 participants remained eligible for regression analysis in Model 1. Further refinement based on demographic criteria resulted in a final cohort of 488,357 individuals for regression analysis in Model 2. This cohort included 222,304 males, representing 45.5% of the total population (Figure 1).

### Associations of albumin with NDDs

**Figure 2:**
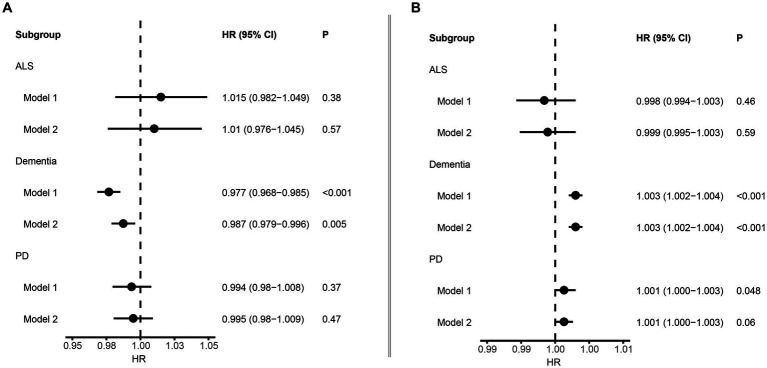
Forest plot showing the association between albumin and neurodegenerative disorders. **(A)** Results from Cox proportional hazards regression and subgroup analyses of the association between serum albumin and neurodegenerative disorders. Error bars indicate 95% confidence intervals. **(B)** Results from Cox proportional hazards regression and subgroup analyses of the association between microalbuminuria and neurodegenerative disorders. Bold *p* value denotes *p* value <0.05.

**Figure 3:**
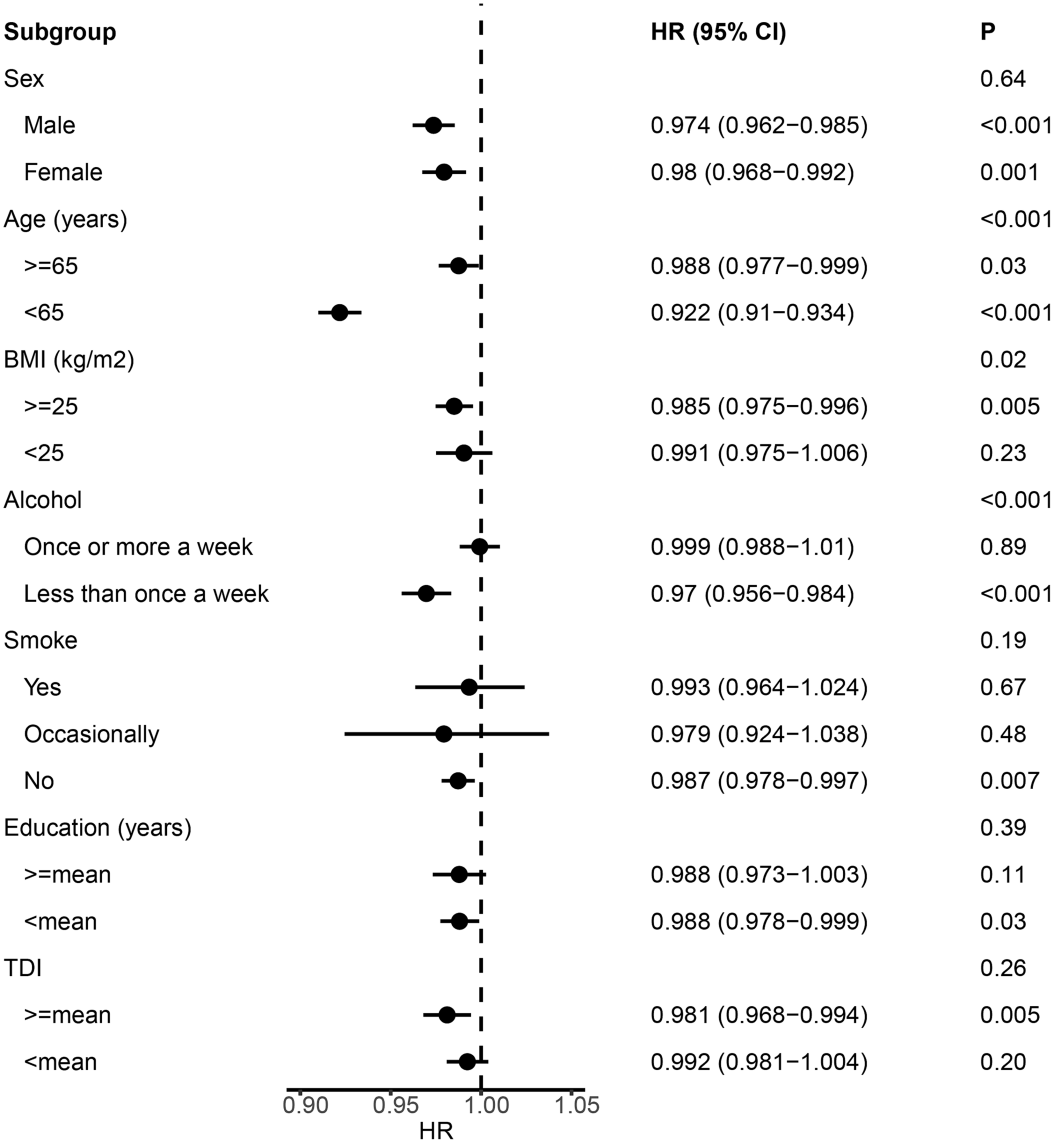
Forest plot showing the results from the subgroup analysis between albumin and dementia. *p* values for each covariate were derived from interaction analysis, while p values for each subgroup were derived from the Cox proportional hazards regression analysis. Error bars indicate 95% confidence intervals.

We employed Cox proportional hazard regression analysis to investigate the longitudinal associations between baseline albumin level and incident NDDs. Over a mean follow-up period of 13.3 years, 9,758 participants developed dementia, after excluding those with dementia at baseline (*N* = 546) or diagnosed within 2 years of initial sampling (*N* = 124). Generally, individuals who developed dementia were older, had a higher body mass index (BMI), lower education levels, and higher Townsend deprivation index scores (Table 1). After adjusting for fundamental covariates in Model 1, each standard deviation (SD) increase in serum albumin was associated with a reduced risk of incident dementia (beta = −0.024, SE = 0.004, *p* = 7.28E-08; Figure 2). This association remained consistent and robust after further adjusting for additional covariates in Model 2 (beta = −0.013, SE = 0.005, *p* = 4.54E-03). Furthermore, significant interactions were observed among age, BMI, alcohol consumption, and serum albumin regarding the risk of incident dementia. Higher serum albumin was found to be a more substantial protective factor in participants with lower alcohol consumption, higher BMI, and younger ages. In subgroup analyses, significant associations were observed across both genders and among individuals with higher BMI, lower alcohol consumption, or younger ages (Figure 3). Conversely, each SD increase in urine microalbumin was associated with a higher risk of incident dementia, though the effect size was modest (beta = 0.003, SE = 3.30E-04, *p* < 0.001).

**Table 1:** Baseline characteristics of the United Kingdom biobank cohort by disease status.

Baseline characteristic	Dementia			ALS			PD		
	Cases	Non-cases	*p*	Cases	Non-cases	*p*	Cases	Non-cases	*p*
Sociodemographic									
Age (years)	64.15 (4.82)	56.37 (8.07)	<0.001	60.73 (6.68)	56.52 (8.10)	<0.001	62.82 (5.38)	56.47 (8.09)	<0.001
Education (years)	12.28 (5.23)	13.99 (5.13)	<0.001	13.41 (5.29)	13.96 (5.13)	0.012	13.38 (5.31)	13.96 (5.13)	<0.001
BMI (kg/m^2^)	27.77 (4.92)	27.42 (4.79)	<0.001	27.35 (4.56)	27.42 (4.80)	0.684	27.77 (4.48)	27.42 (4.80)	<0.001
Townsend deprivation index	−1.00 (3.27)	−1.33 (3.07)	<0.001	−1.42 (3.03)	−1.32 (3.08)	0.437	−1.46 (3.01)	−1.32 (3.08)	0.010
Smoke: No	0.90 (8271)	0.90 (428490)	0.918	0.90 (538)	0.90 (436608)	0.789	0.94 (3202)	0.89 (433129)	<0.001
Occasionally	0.02 (199)	0.03 (13114)	<0.001	0.03 (15)	0.03 (13310)	0.900	0.02 (58)	0.03 (13247)	<0.001
Mostly	0.08 (762)	0.08 (36885)	0.054	0.08 (45)	0.08 (37719)	0.939	0.05 (162)	0.08 (37575)	<0.001
Alcohol: Never	0.13 (1230)	0.08 (37335)	<0.001	0.07 (44)	0.08 (38638)	0.705	0.11 (370)	0.08 (38177)	<0.001
Special occasions	0.14 (1288)	0.11 (54617)	<0.001	0.12 (69)	0.11 (55901)	0.949	0.11 (384)	0.11 (55471)	0.686
1–3 times a month	0.10 (926)	0.11 (53534)	<0.001	0.11 (65)	0.11 (54436)	0.897	0.10 (326)	0.11 (54091)	0.002
1–2 times a week	0.23 (2112)	0.26 (123932)	<0.001	0.27 (162)	0.26 (125952)	0.483	0.24 (810)	0.26 (125105)	0.004
3–4 times a week	0.19 (1768)	0.23 (111292)	<0.001	0.20 (122)	0.23 (113013)	0.110	0.22 (744)	0.23 (112236)	0.046
Daily	0.21 (1908)	0.20 (97779)	0.584	0.23 (136)	0.20 (99697)	0.171	0.23 (788)	0.20 (98871)	<0.001
Biomarker									
Serum albumin (g/L)	44.70 (2.72)	45.22 (2.62)	<0.001	45.17 (2.48)	45.21 (2.63)	0.689	44.92 (2.63)	45.21 (2.63)	<0.001
Urine microalbumin (mg/L)	28.02 (47.40)	21.89 (35.83)	<0.001	20.69 (29.29)	22.05 (36.20)	0.489	25.43 (43.20)	22.03 (36.14)	0.006

Continuous variables were presented as median (SD); Categorical variables were presented as percentages (numbers).

In addition, a total of 3,544 patients with PD and 628 patients with ALS were identified during the follow-up. Generally, patients with PD were older, had lower education level, larger BMI and lower Townsend deprivation index, and tended to smoke less, while patients with ALS were older and had smaller BMI (Table 1). However, no significant association was identified between albumin level and risk of ALS and PD (Figure 2).

## Discussion

Previous clinical and epidemiological studies have proposed a potential link between albumin levels and the risk of NDDs. However, these findings have been inconsistent and lack a comprehensive systemic investigation. In the present study, we examined the relationship between baseline albumin levels and NDD risk using data from the large prospective United Kingdom Biobank cohort. Our analysis demonstrated that higher serum albumin levels are associated with a reduced risk of dementia. These findings enhance our understanding of albumin’s role in NDD risk and suggest that targeting albumin could be a promising strategy for future clinical trials focused on dementia treatment.

Albumin, the predominant protein in blood plasma, plays essential roles in maintaining blood volume, transporting hormones, vitamins, and enzymes throughout the body, and ensuring proper fluid distribution within blood vessels. However, when the blood–brain barrier is compromised, albumin can infiltrate brain tissue, potentially triggering neurodegeneration (16). AD is the most prevalent form of dementia characterized by extracellular deposition of β-amyloid protein (Aβ) and intraneuronal neurofibrillary tangles in the brain. Several endogenous proteins interacting with Aβ can modulate its amyloidogenic process, including serum albumin, a key endogenous inhibitor of Αβ aggregation (17). Serum albumin binds to Aβ and facilitates Aβ efflux from the cerebrospinal fluid to plasma. A previous seven-year retrospective cohort study among 1,800 subjects showed that relatively low serum albumin levels at baseline (< 40.5 g/L) were associated with an increased risk of mild cognitive impairment (HR: 2.18, 95% CI: 1.67–2.82) (7). Similarly, another retrospective cohort study among 2,396 Koreans suggested sustained lower serum albumin levels were associated with lower cognitive performance measured by the Mini-Mental State Examination scores (18). Cohort studies have also suggested that low serum albumin is associated with increased odds of cognitive impairment (19, 20) and AD (9, 21) in the elderly population. However, it was also reported that serum albumin concentrations did not differ according to cognitive function in 540 older cognitively normal adults from the single-center Conselice Study of Brain Aging (8). Nevertheless, the sample sizes of these studies were relatively small. In the current study, our results suggested that higher serum albumin level at baseline was associated with a decreased risk of dementia. From the pathogenesis perspective, previous study showed that serum albumin level was inversely associated with Aβ deposition and Aβ positivity (9). A reduction in Aβ binding to serum albumin in the blood may lead to a decrease in the capacity for Aβ excretion from the brain to the blood, resulting in Aβ deposition in the brain. Low serum albumin results in less binding to Aβ in blood, which increases blood plasma Aβ concentration, resulting in increased Aβ deposition in the brain by blocking the Aβ shift due to its lower concentration difference. Therefore, serum albumin plays an important role in the Aβ shift from the brain to the blood plasma for balancing the dynamic equilibrium of Aβ between the brain and blood. Meanwhile, the chaperone-like activity of serum albumin suggests it protects against protein misfolding and aggregation (22), which is closely implicated in the pathogenesis of NDDs. In addition, albumin serves as an antioxidant which may help prevent excessive oxidant stress induced by inflammation in the aging neuronal cell (23). Experimental studies suggested that inflammatory mechanisms are involved in the pathogenesis of dementia, and brain atrophy and cognitive decline in AD may be triggered by acute and chronic systemic inflammation. Considering antioxidants’ ability to reduce inflammatory responses, the beneficial effect of albumin, as a primary transporter and extracellular antioxidant in the human body, on cognitive function appears biologically plausible. In addition, plasma exchange with albumin replacement was shown to slow cognitive and functional decline in AD (24), suggesting the therapeutic effect of albumin in dementia. Meanwhile, there has been comprehensive progress of albumin-based drug delivery for the treatment of AD based on biological potency such as Aβ disaggregation (25). For example, serum albumin-manganese dioxide nanocomposites and silibinin-albumin nanoparticles have been reported in AD treatment with efficacy (26, 27). These findings underscore the potential clinical utility of serum albumin as a biomarker for both diagnosis and prognosis prediction in dementia, and suggested the potential for targeting albumin to treat dementia in clinical trials.

In addition, our results revealed that higher level of albumin in urine was associated with a higher risk of dementia. Consistently, albuminuria was identified as a significant risk factor for the development of dementia, including AD in a longitudinal study among 1,562 community-dwelling Japanese subjects aged over 60 years without dementia (28). Similar results were observed in another community-based cohort study in Taiwan (29). Albuminuria, an early manifestation of chronic kidney disease and a marker of endothelial dysfunction and vascular risk, remains less studied in terms of the exact mechanisms underlying its association with dementia. The increased permeability of various plasma proteins and impaired clearance of amyloid β at the blood–brain barrier due to endothelial dysfunction may contribute to this association. Clinical trials are imperative to determine whether interventions targeting albuminuria can prevent cognitive decline among older individuals with this early marker of kidney disease. However, it is noteworthy that microalbuminuria was also reported not to be associated with subsequent cognitive decline and incident dementia (30). Additionally, the effect size identified in our study was limited. Consequently, further research is necessary to comprehensively explore the association between albumin in urine and the risk of dementia. Previous epidemiological studies have also suggested association between albumin and risk of PD and ALS. So we also examined the association between baseline albumin levels and the risk of PD and ALS, but did not find significant associations. However, these results should be interpreted with caution due to the relatively small number of patients identified during the follow-up period. Consequently, further research is needed to explore these associations more comprehensively.

The current study investigated the impact of baseline albumin levels on the risk of developing NDDs. One of the key strengths of this study is its use of longitudinal data from a large cohort, which allows for robust analysis of the association between baseline albumin levels and NDD risk. Additionally, the extensive and detailed clinical data available enabled us to account for potential confounding factors and conduct a series of sensitivity and stratification analyses to validate our findings. However, several important limitations must be acknowledged. Firstly, the study cohort predominantly consisted of Caucasian individuals, which may limit the generalizability of the results to other ethnic populations. Future research should aim to include diverse ancestral cohorts to validate and extend these findings. Secondly, the effect size of urine microalbumin on dementia risk was relatively modest, suggesting that its influence may be limited in this context. Thirdly, the study focused solely on albumin levels in serum and urine. Considering the etiology of NDDs, examining albumin levels in cerebrospinal fluid (CSF) could provide novel insights. Fourthly, our study only analyzed albumin levels without considering additional biomarkers. Some additional biomarkers such as tau and beta-amyloid were also shown to be associated with risk of NDDs. Further exploration of other biomarkers in conjunction with albumin could offer a more comprehensive understanding and aid in the screening of NDDs. Lastly, our analysis focused on all-cause dementia. Further research examining specific subtypes of dementia could yield additional insights into the role of albumin in different forms of dementia.

## Conclusion

Our study findings suggest that higher pre-existing serum albumin levels are associated with a reduced risk of developing dementia, while elevated urine microalbumin levels are linked to an increased risk of dementia. These results propose that albumin may serve as a valuable prognostic biomarker for dementia. Gaining a deeper understanding of the underlying mechanisms could pave the way for novel therapeutic approaches targeting albumin, such as albumin-manganese dioxide nanocomposites and silibinin-albumin nanocomposites. These advancements could enable clinicians to intervene more effectively, potentially slowing or delaying the progression of dementia.

## Data Availability

The original contributions presented in the study are included in the article/Supplementary material, further inquiries can be directed to the corresponding author/s.

## Footnotes

^1^ https://biobank.ndph.ox.ac.uk/ukb/ukb/docs/serum_biochemistry.pdf
