# Supplementary material for: Protective role of serum albumin in dementia: a prospective study from United Kingdom biobank
Source: Front Neurol. 2024 Aug 14;15:1458184. doi: 10.3389/fneur.2024.1458184 (PMC11349656; doi:10.3389/fneur.2024.1458184)
Supplement: Supplementary file 1 [file Data_Sheet_1.DOCX]

**Supplementary Table 1. ICD-10 and read codes used to identify patients with Parkinson’s disease.**

| **Disease** | **ICD-10 code** | **self-reported disease code** | **Read code V2** | **Read code V3** |
| --- | --- | --- | --- | --- |
| Dementia | A810, F00, F000, F001, F002, F009, F01, F010, F011, F012, F013, F018, F019, F02, F020, F021, F022, F023, F024, F028, F03, F051, F106, G30, G300, G301, G308, G309, G310, G311, G318, I673 | 1263 | A411., A4110, Eu01., Eu010, Eu011, Eu012, Eu013, Eu01y, Eu01z, Eu02., Eu041, Eu106, Fyu30, F1101, Fyu30, F110., F111., F112., F11y., F10y., F10y0, F10y1, F10y2, F10yz, F118., F11y2, F11yz, Fyu31, I673 | 1B1A0, A411., E0011, E003., E0040, E0040, E0041, E0041, E0041, E0042, E0042, E0043, E0043, E011., E011., E0111, E0112, E011z, E02y2, E031., Eu01y, Eu01z, Eu041, Eu106, F10y., F10y0, F10y1, F10yz, F110., F110., F111., F112., F11x7, F11yz, F21y2, Fyu30, Fyu31, X002U, X002V, X002W, X002w, X002y, X002z, X0031, X0032, X0033, X0035, X0036, X0037, X0039, X003E, X003F, X003G, X003K, X003R, X003T, X003V, X003W, X003X, X003m, X004B, X004E, X005L, X005M, X005N, X005O, X005P, X00Qz, X00R2, X00RH, XE1Xs, XE1Xs, XE1Xv, XE1YQ, XM09N, XM09O, Xa0lH, Xa0lH, Xa0sC, XaA1S, XaIKB, XaIKC, XaIRJ, XaPws, XabVp |
| Parkinson’s disease | G20 | 1262 | F12.., F120., F12z., 147F. | F12.., F120., F12z., .F22., .F22Z, .F221, 147F., X003a, XaQwf |
| Amyotrophic lateral sclerosis | G122 | 1259 | F152., F1520, F152z | .F251, F1520, F152z, F152. |
